# Crystal structure of a zigzag Co^II^ coordination polymer: *catena*-poly[[di­chlorido­bis­(methanol-κ*O*)cobalt(II)]-μ-bis­(pyridin-3-ylmeth­yl)sulfane-κ^2^ *N*:*N*′]

**DOI:** 10.1107/S2056989017016449

**Published:** 2017-11-17

**Authors:** Suk-Hee Moon, Joobeom Seo, Ki-Min Park

**Affiliations:** a Department of Food and Nutrition, Kyungnam College of Information and Technology, Busan 47011, Republic of Korea; b Mineral Resources Research Division, Korea Institute of Geoscience and Mineral Resources (KIGAM), Daejeon 34132, Republic of Korea; c Research institute of Natural Science, Gyeongsang National University, Jinju 52828, Republic of Korea

**Keywords:** crystal structure, cobalt(II), dipyridyl ligand, chloride anion, zigzag chain, hydrogen bonding

## Abstract

Reaction of bis­(pyridin-3-ylmeth­yl)sulfane (*L*) with cobalt(II) chloride in methanol led to the formation of the title coordination polymer, [CoCl_2_(C_12_H_12_N_2_S)(CH_3_OH)_2_]_*n*_, in which the Co^II^ cation lies on a crystallographic inversion centre and the S atom of the *L* ligand lies on a twofold rotation axis. Each Co^II^ ion is coordinated by two pyridine N atoms from two bridging *L* ligands, two O atoms from methanol mol­ecules and two chloride anions, all inversion-related. The complex unit has an elongated octa­hedral geometry, in which N_2_O_2_ donor atoms occupy the equatorial positions and two chloride anions occupy the axial positions. Each *L* ligand links two Co^II^ ions, forming an infinite zigzag chain propagating along the *c-*axis direction and further stabilized by O—H⋯Cl hydrogen bonds between the methanol mol­ecules and the chloride anions. Adjacent chains in the structure are connected by inter­molecular C—H⋯Cl hydrogen bonds, resulting in the formation of a three-dimensional supra­molecular architecture.

## Abstract 2

In the title compound, each Co^II^ ion is coordinated by two pyridine N atoms from two individual dipyridyl ligands, two methanol O atoms and two chloride anions in an elongated octa­hedral geometry. Each dipyridyl ligand links two Co^II^ ions, forming infinite zigzag chains.

## Chemical context   

Up to now, large numbers of metal–organic coordination polymers with intriguing topologies and attractive properties have been constructed in which dipyridyl-type mol­ecules functioning as bridging ligands have mainly been used (Leong & Vittal, 2011 ▸; Wang *et al.*, 2012 ▸; Liu *et al.*, 2011 ▸). Our group has also investigated several metal–organic coordination polymers with inter­esting topologies using such dipyridyl-type ligands (Moon *et al.*, 2011 ▸, 2016 ▸, 2017 ▸; Lee *et al.*, 2015 ▸; Ju *et al.*, 2014 ▸; Im *et al.*, 2017 ▸). In an extension of our research in this area, the title compound was prepared by the reaction of cobalt(II) chloride with bis­(pyridin-3-ylmeth­yl)sulfane, C_12_H_12_N_2_S, (*L*) as a flexible dipyridyl-type ligand [synthesized according to a literature procedure (Park *et al.*, 2010 ▸; Lee *et al.*, 2012 ▸)]. Our group has previously reported the crystal structure of a looped-chain Co^II^ coordination polymer obtained through the reaction of cobalt(II) nitrate with *L* (Moon *et al.*, 2017 ▸). In this article, we describe the crystal structure of the title compound, [Co(*L*)(CH_3_OH)_2_Cl_2_]_*n*_, with *L* = (pyridin-3-ylmeth­yl)sulfane, which adopts a one-dimensional zigzag topology.
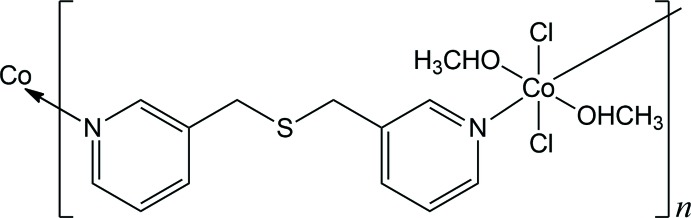


## Structural commentary   

**Figure 1:**
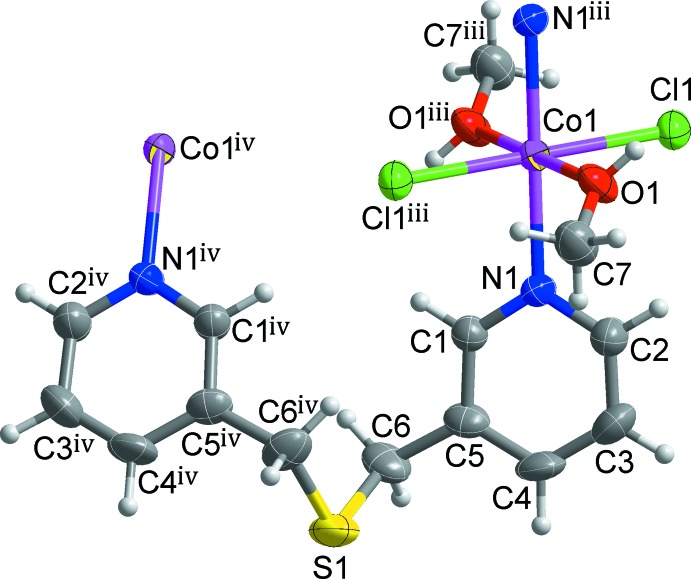
A view of mol­ecular structure of the title compound, showing the geometry around the Co^II^ centre and the atom-numbering scheme [symmetry codes: (iii) −*x* + 1, −*y*, −*z*; (iv) −*x* + 1, *y*, −*z* − 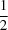]. Displacement ellipsoids are drawn at the 50% probability level. H atoms are shown as small spheres of arbitrary radius.

As shown in Figs. 1 ▸ and 2 ▸, the mol­ecular structure of the title compound is generated by the combination of inversion and twofold rotation symmetries. Each Co^II^ cation lies on a crystallographic inversion centre, and the twofold rotation axis passes through the S atom of the *L* ligand. Therefore, the asymmetric unit comprises one half of a Co^II^ cation, one half of an *L* ligand, one chloride anion and one methanol mol­ecule. The coordination geometry of the Co^II^ ion is elongated octa­hedral with the four equatorial positions being occupied by two pyridine N atoms from the two symmetry-related *L* ligands and two O atoms from two symmetry-related methanol mol­ecules, and the two axial positions being occupied by two chlorido ligands (Fig. 1 ▸). Selected bond lengths and angles around the Co1 atom are listed in Table 1 ▸. The coordination geometry of the title compound is similar to that found in di­chloro­bis­(methanol-κ*O*)bis­[*N*-(1-naphth­yl)-*N*′-(3-pyrid­yl)urea-κ*N*]cobalt(II) (Huang *et al.*, 2008 ▸).

**Figure 2:**
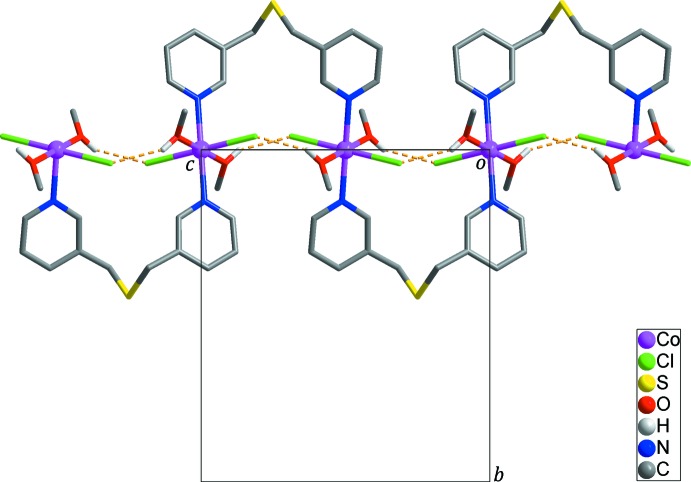
The zigzag chain structure of the title compound extending along the *c* axis. Yellow dashed lines represent intra­molecular O—H⋯Cl hydrogen bonds in the zigzag chain. H atoms not involved in inter­molecular inter­actions have been omitted for clarity.

Each *L* ligand bridges two Co^II^ ions into an infinite zigzag chain propagating along the *c-*axis direction (Fig. 2 ▸). The separation between the Co^II^ ions through a *L* ligand in the chain is 6.0595 (11) Å. The flexible thio­ether segment [C5—C6—S1—C6^iv^—C5^iv^; symmetry code: (iv) −*x* + 1, *y*, −*z* − 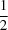] of the *L* ligand shows a bent arrangement induced by a *gauche*–*gauche* configuration with a torsion angle of 74.4 (3)° for the C5—C6—S1—C6^iv^ and C6—S1—C6^iv^—C5^iv^ units. This conformation of the *L* ligand is similar to those in a cyclic dimer-type silver(I) BF_4_ complex, [Ag(*L*)]_2_·2BF_4_ (Seo *et al.*, 2003 ▸), and a staircase-type copper(I) iodide coordination polymer, [(CuI)_2_(*L*)]_*n*_ (Hanton *et al.*, 2006 ▸). The zigzag topology of the chain may be induced by this conformation of the *L* ligand.

## Supra­molecular features   

**Figure 3:**
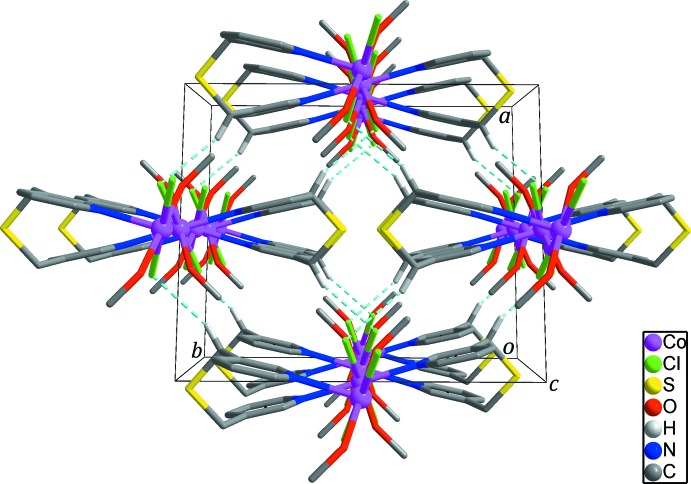
The three-dimensional supra­molecular network formed through inter­molecular C—H⋯Cl hydrogen bonds (sky-blue dashed lines) between the chains. H atoms not involved in inter­molecular inter­actions have been omitted for clarity.

An O1—H⋯Cl1^i^ hydrogen bond (Table 2 ▸; yellow dashed lines in Fig. 2 ▸; symmetry code: (i) −*x* + 1, *y*, −*z* + 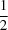) between the methanol mol­ecule and the chloride anion contributes to the stabilization of the zigzag chain. In the crystal, weak C6—H⋯Cl1^ii^ hydrogen bonds (Table 2 ▸; sky-blue dashed lines in Fig. 3 ▸) connect neighboring zigzag chains to generate a three-dimensional supra­molecular network.

## Database survey   

A search of the Cambridge Structural Database (Version 5.38, update May 2017; Groom *et al.*, 2016 ▸) for the title ligand (*L*) gave three hits. Two of these (REJCAL, RENHOI; Hanton *et al.*, 2006 ▸) are copper(I) iodide coordination polymers adopting staircase- and loop-type structures, respectively. The third (EXEZOW; Seo *et al.*, 2003 ▸) is a silver(I) BF_4_ complex with a cyclic dimer structure.

## Synthesis and crystallization   

The *L* ligand was synthesized according to a literature method (Park *et al.*, 2010 ▸; Lee *et al.*, 2012 ▸). Crystals of the title compound were obtained by slow evaporation of a methanol solution of the *L* ligand with CoCl_2_·6H_2_O in an 1:1 molar ratio.

## Refinement   

Crystal data, data collection and structure refinement details are summarized in Table 3 ▸. All H atoms were positioned geometrically with C—H = 0.93 Å for C*sp* ^2^—H, 0.96 Å for methyl C—H, 0.97 Å for methyl­ene C—H, and 0.82 Å for alcohol O—H, and were refined as riding with *U*_iso_(H) = 1.2*U*_eq_(C,O).

## Supplementary Material

Crystal structure: contains datablock(s) I, New_Global_Publ_Block. DOI: 10.1107/S2056989017016449/zs2393sup1.cif

Structure factors: contains datablock(s) I. DOI: 10.1107/S2056989017016449/zs2393Isup2.hkl

CCDC reference: 1585746

Additional supporting information:  crystallographic information; 3D view; checkCIF report

## Figures and Tables

**Table 1:** Selected geometric parameters (Å, °)

Co1—O1	2.119 (2)	Co1—Cl1	2.4571 (11)
Co1—N1	2.153 (3)		
			
O1—Co1—N1	88.20 (11)	N1—Co1—Cl1	92.11 (9)
O1—Co1—Cl1	90.53 (8)		

**Table 2:** Hydrogen-bond geometry (Å, °)

*D*—H⋯*A*	*D*—H	H⋯*A*	*D*⋯*A*	*D*—H⋯*A*
O1—H1*A*⋯Cl1^i^	0.82	2.28	3.030 (3)	152
C6—H6*A*⋯Cl1^ii^	0.97	2.76	3.653 (4)	153

Symmetry codes: (i) 
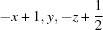
; (ii) 
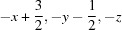
.

**Table 3:** Experimental details

Crystal data	
Chemical formula	[CoCl_2_(C_12_H_12_N_2_S)(CH_4_O)_2_]
*M*_r_	410.21
Crystal system, space group	Monoclinic, *C*2/*c*
Temperature (K)	298
*a*, *b*, *c* (Å)	11.419 (2), 13.363 (2), 12.119 (2)
β (°)	106.226 (4)
*V* (Å^3^)	1775.6 (6)
*Z*	4
Radiation type	Mo *K*α
μ (mm^−1^)	1.39
Crystal size (mm)	0.35 × 0.08 × 0.08

Data collection	
Diffractometer	Bruker CCD area detector
Absorption correction	Multi-scan (*SADABS*; Bruker, 2014 ▸)
*T*_min_, *T*_max_	0.627, 0.888
No. of measured, independent and observed [*I* > 2σ(*I*)] reflections	5461, 1947, 1013
*R*_int_	0.098
(sin θ/λ)_max_ (Å^−1^)	0.639

Refinement	
*R*[*F* ^2^ > 2σ(*F* ^2^)], *wR*(*F* ^2^), *S*	0.047, 0.095, 0.92
No. of reflections	1947
No. of parameters	102
H-atom treatment	H-atom parameters constrained
Δρ_max_, Δρ_min_ (e Å^−3^)	0.37, −0.32

Computer programs: *APEX2* and *SAINT* (Bruker, 2014 ▸), *SHELXS97* and *SHELXTL* (Sheldrick, 2008 ▸), *SHELXL2014* (Sheldrick, 2015 ▸), *DIAMOND* (Brandenburg, 2010 ▸) and *publCIF* (Westrip, 2010 ▸).

## supplementary crystallographic information

### Crystal data

**Table:**

[CoCl_2_(C_12_H_12_N_2_S)(CH_4_O)_2_]	*F*(000) = 844
*M**_r_* = 410.21	*D*_x_ = 1.534 Mg m^−^^3^
Monoclinic, *C*2/*c*	Mo *K*α radiation, λ = 0.71073 Å
*a* = 11.419 (2) Å	Cell parameters from 5771 reflections
*b* = 13.363 (2) Å	θ = 2.4–28.2°
*c* = 12.119 (2) Å	µ = 1.39 mm^−^^1^
β = 106.226 (4)°	*T* = 298 K
*V* = 1775.6 (6) Å^3^	Needle, violet
*Z* = 4	0.35 × 0.08 × 0.08 mm

### Data collection

**Table:**

Bruker CCD area detector diffractometer	1013 reflections with *I* > 2σ(*I*)
φ and ω scans	*R*_int_ = 0.098
Absorption correction: multi-scan (SADABS; Bruker, 2014)	θ_max_ = 27.0°, θ_min_ = 2.4°
*T*_min_ = 0.627, *T*_max_ = 0.888	*h* = −14→14
5461 measured reflections	*k* = −17→13
1947 independent reflections	*l* = −15→15

### Refinement

**Table:**

Refinement on *F*^2^	0 restraints
Least-squares matrix: full	Hydrogen site location: inferred from neighbouring sites
*R*[*F*^2^ > 2σ(*F*^2^)] = 0.047	H-atom parameters constrained
*wR*(*F*^2^) = 0.095	*w* = 1/[σ^2^(*F*_o_^2^) + (0.0319*P*)^2^] where *P* = (*F*_o_^2^ + 2*F*_c_^2^)/3
*S* = 0.92	(Δ/σ)_max_ < 0.001
1947 reflections	Δρ_max_ = 0.37 e Å^−^^3^
102 parameters	Δρ_min_ = −0.32 e Å^−^^3^

### Special details

**Table:**

Geometry. All esds (except the esd in the dihedral angle between two l.s. planes) are estimated using the full covariance matrix. The cell esds are taken into account individually in the estimation of esds in distances, angles and torsion angles; correlations between esds in cell parameters are only used when they are defined by crystal symmetry. An approximate (isotropic) treatment of cell esds is used for estimating esds involving l.s. planes.

### Fractional atomic coordinates and isotropic or equivalent isotropic displacement parameters (Å^2^)

**Table:**

	*x*	*y*	*z*	*U*_iso_*/*U*_eq_	
Co1	0.5000	0.0000	0.0000	0.0333 (2)	
Cl1	0.64563 (9)	0.04038 (8)	0.18588 (8)	0.0414 (3)	
S1	0.5000	−0.44202 (11)	−0.2500	0.0611 (5)	
O1	0.3665 (2)	−0.0394 (2)	0.0836 (2)	0.0467 (8)	
H1A	0.3528	−0.0010	0.1313	0.056*	
N1	0.5559 (3)	−0.1544 (2)	0.0120 (3)	0.0361 (8)	
C1	0.5742 (3)	−0.2051 (3)	−0.0774 (3)	0.0365 (10)	
H1	0.5682	−0.1696	−0.1448	0.044*	
C2	0.5680 (4)	−0.2068 (3)	0.1089 (4)	0.0467 (12)	
H2	0.5578	−0.1734	0.1729	0.056*	
C3	0.5946 (4)	−0.3071 (4)	0.1189 (4)	0.0599 (14)	
H3	0.6021	−0.3408	0.1877	0.072*	
C4	0.6099 (4)	−0.3567 (3)	0.0235 (4)	0.0562 (13)	
H4	0.6262	−0.4250	0.0274	0.067*	
C5	0.6011 (4)	−0.3051 (3)	−0.0771 (4)	0.0416 (11)	
C6	0.6204 (4)	−0.3550 (3)	−0.1819 (4)	0.0543 (13)	
H6A	0.6975	−0.3907	−0.1601	0.065*	
H6B	0.6260	−0.3040	−0.2372	0.065*	
C7	0.2751 (4)	−0.1150 (3)	0.0567 (4)	0.0570 (13)	
H7A	0.2241	−0.1053	−0.0202	0.068*	
H7B	0.2265	−0.1112	0.1096	0.068*	
H7C	0.3132	−0.1796	0.0625	0.068*	

### Atomic displacement parameters (Å^2^)

**Table:**

	*U*^11^	*U*^22^	*U*^33^	*U*^12^	*U*^13^	*U*^23^
Co1	0.0461 (5)	0.0281 (5)	0.0281 (4)	0.0030 (4)	0.0143 (4)	0.0000 (4)
Cl1	0.0512 (7)	0.0429 (6)	0.0305 (6)	0.0051 (5)	0.0122 (5)	0.0002 (4)
S1	0.0791 (14)	0.0273 (10)	0.0658 (13)	0.000	0.0021 (11)	0.000
O1	0.064 (2)	0.0387 (17)	0.0454 (18)	−0.0111 (15)	0.0286 (17)	−0.0149 (13)
N1	0.048 (2)	0.029 (2)	0.034 (2)	0.0012 (16)	0.0140 (18)	0.0029 (16)
C1	0.039 (3)	0.035 (3)	0.035 (2)	0.004 (2)	0.010 (2)	0.000 (2)
C2	0.061 (3)	0.041 (3)	0.039 (3)	0.006 (2)	0.016 (2)	0.002 (2)
C3	0.080 (4)	0.047 (3)	0.050 (3)	0.005 (3)	0.013 (3)	0.020 (3)
C4	0.072 (4)	0.025 (3)	0.067 (4)	0.010 (2)	0.012 (3)	0.010 (2)
C5	0.034 (3)	0.033 (3)	0.056 (3)	0.006 (2)	0.011 (2)	−0.003 (2)
C6	0.052 (3)	0.045 (3)	0.064 (3)	0.015 (2)	0.015 (3)	−0.014 (2)
C7	0.068 (3)	0.054 (3)	0.052 (3)	−0.013 (3)	0.022 (3)	−0.008 (2)

### Geometric parameters (Å, º)

**Table:**

Co1—O1	2.119 (2)	C1—H1	0.9300
Co1—O1^i^	2.119 (2)	C2—C3	1.372 (6)
Co1—N1	2.153 (3)	C2—H2	0.9300
Co1—N1^i^	2.153 (3)	C3—C4	1.385 (6)
Co1—Cl1^i^	2.4571 (11)	C3—H3	0.9300
Co1—Cl1	2.4571 (11)	C4—C5	1.380 (6)
S1—C6	1.814 (4)	C4—H4	0.9300
S1—C6^ii^	1.814 (4)	C5—C6	1.504 (5)
O1—C7	1.424 (4)	C6—H6A	0.9700
O1—H1A	0.8200	C6—H6B	0.9700
N1—C2	1.342 (5)	C7—H7A	0.9600
N1—C1	1.343 (5)	C7—H7B	0.9600
C1—C5	1.371 (5)	C7—H7C	0.9600
			
O1—Co1—O1^i^	180.0	N1—C2—C3	123.6 (4)
O1—Co1—N1	88.20 (11)	N1—C2—H2	118.2
O1^i^—Co1—N1	91.80 (11)	C3—C2—H2	118.2
O1—Co1—N1^i^	91.80 (11)	C2—C3—C4	118.0 (4)
O1^i^—Co1—N1^i^	88.20 (11)	C2—C3—H3	121.0
N1—Co1—N1^i^	180.0	C4—C3—H3	121.0
O1—Co1—Cl1^i^	89.47 (8)	C5—C4—C3	120.3 (4)
O1^i^—Co1—Cl1^i^	90.53 (8)	C5—C4—H4	119.9
N1—Co1—Cl1^i^	87.89 (9)	C3—C4—H4	119.9
N1^i^—Co1—Cl1^i^	92.11 (9)	C1—C5—C4	116.7 (4)
O1—Co1—Cl1	90.53 (8)	C1—C5—C6	121.0 (4)
O1^i^—Co1—Cl1	89.47 (8)	C4—C5—C6	122.2 (4)
N1—Co1—Cl1	92.11 (9)	C5—C6—S1	113.4 (3)
N1^i^—Co1—Cl1	87.89 (9)	C5—C6—H6A	108.9
Cl1^i^—Co1—Cl1	180.0	S1—C6—H6A	108.9
C6—S1—C6^ii^	100.3 (3)	C5—C6—H6B	108.9
C7—O1—Co1	130.2 (2)	S1—C6—H6B	108.9
C7—O1—H1A	109.5	H6A—C6—H6B	107.7
Co1—O1—H1A	119.1	O1—C7—H7A	109.5
C2—N1—C1	116.2 (4)	O1—C7—H7B	109.5
C2—N1—Co1	121.1 (3)	H7A—C7—H7B	109.5
C1—N1—Co1	122.6 (3)	O1—C7—H7C	109.5
N1—C1—C5	125.1 (4)	H7A—C7—H7C	109.5
N1—C1—H1	117.5	H7B—C7—H7C	109.5
C5—C1—H1	117.5		
			
C2—N1—C1—C5	1.6 (6)	N1—C1—C5—C6	−179.8 (4)
Co1—N1—C1—C5	−175.0 (3)	C3—C4—C5—C1	−1.4 (7)
C1—N1—C2—C3	−1.6 (6)	C3—C4—C5—C6	178.3 (4)
Co1—N1—C2—C3	175.1 (3)	C1—C5—C6—S1	−110.7 (4)
N1—C2—C3—C4	0.1 (7)	C4—C5—C6—S1	69.6 (5)
C2—C3—C4—C5	1.4 (7)	C6^ii^—S1—C6—C5	74.4 (3)
N1—C1—C5—C4	−0.1 (6)		

Symmetry codes: (i) −*x*+1, −*y*, −*z*; (ii) −*x*+1, *y*, −*z*−1/2.

### Hydrogen-bond geometry (Å, º)

**Table:**

*D*—H···*A*	*D*—H	H···*A*	*D*···*A*	*D*—H···*A*
O1—H1*A*···Cl1^iii^	0.82	2.28	3.030 (3)	152
C6—H6*A*···Cl1^iv^	0.97	2.76	3.653 (4)	153

Symmetry codes: (iii) −*x*+1, *y*, −*z*+1/2; (iv) −*x*+3/2, −*y*−1/2, −*z*.
